# Comparison of Albumin-Bilirubin, Platelet-Albumin-Bilirubin, and Child-Pugh scores to predict overall survival in patients with stage C hepatocellular carcinoma with liver cirrhosis treated with c-TACE

**DOI:** 10.3389/fonc.2025.1613697

**Published:** 2025-08-27

**Authors:** Fei Wu, Zhihong Zhang, Shanshan Liu

**Affiliations:** ^1^ Department of Thyroid Surgery, The Affiliated Hospital of Southwest Medical University, Luzhou, China; ^2^ Department of General Medicine, The Affiliated Hospital of Southwest Medical University, Luzhou, China

**Keywords:** hepatocellular carcinoma, liver cirrhosis, ALBI, PALBI, Child-Pugh, c-TACE

## Abstract

**Purpose:**

This study aimed to evaluate and compare the prognostic performance of Albumin-Bilirubin (ALBI), Platelet-Albumin-Bilirubin (PALBI), and Child-Pugh (CP) scores in predicting overall survival (OS) among patients with stage C hepatocellular carcinoma (HCC) with liver cirrhosis undergoing conventional transcatheter arterial chemoembolization (c-TACE).

**Methods:**

We performed a retrospective cohort analysis of 151 cirrhotic patients with Barcelona Clinic Liver Cancer (BCLC) stage C HCC treated with c-TACE between 2017 and 2021. Pretreatment CP scores, ALBI, and PALBI were recorded, and their associations with OS were analyzed using Kaplan-Meier methods and receiver operating characteristic (ROC) curve analysis. Multivariate Cox models were used to determine independent survival predictors.

**Results:**

The median OS for the entire study cohort was 18.8 months (95% CI: 11.294-26.306 months). CP score, ALBI grade and PALBI grade were significantly correlated with OS (all P<0.05). Survival analysis revealed dose-dependent relationships: CP classes A and B demonstrated median OS of 22.9 vs 9.9 months (P<0.05), ALBI grades 1–3 corresponded to median OS of 25.7, 12.7, and 3.8 months respectively (P<0.05), PALBI grades 1–3 showed median OS of 25.7, 10.1, and 8.5 months (P<0.05). The area under the receiver operating characteristic curve (AUROC) values of CP score, ALBI grading, ALBI score, PALBI grading and PALBI score were 0.572, 0.550, 0.595, 0.619 and 0.637, respectively. Compared with the CP score and ALBI, PALBI showed a better predictive effect in patients with stage C hepatocellular carcinoma with cirrhosis treated with c-TACE. Multivariate analysis showed that CP grade, ALBI grade, PALBI grade, Portal vein invasion, and the number of c-TACE were independent predictors affecting the survival period of patients.

**Conclusion:**

In cirrhotic patients with BCLC stage C HCC undergoing c-TACE, the PALBI scoring system emerged as a more reliable prognostic indicator than CP and ALBI assessments, showing enhanced discriminative capacity for survival outcomes.

## 1 Introduction

Hepatocellular carcinoma (HCC) is the fourth leading cause of cancer-related death worldwide (1). The prognosis for HCC is often poor because many patients are diagnosed at an advanced stage. According to the current HCC practice guidelines (2, 3), surgical resection, local ablation, and transplantation are recommended for early HCC. Conventional transcatheter arterial chemoembolization (c-TACE) or systemic therapy is recommended for patients with advanced stage (4, 5).

Treatment decisions and prognosis prediction for HCC patients are based on patients’ physical function status, liver function and tumor burden (3). Therefore, liver function evaluation is of great significance in the treatment of HCC. The Child-Pugh scale is a widely used scale for assessing liver function reserve (6). In addition, albumin-bilirubin (ALBI) grading and platelet-albumin-bilirubin (PALBI) grading can also be used to evaluate liver function in HCC patients (7). PALBI grading is developed on the basis of ALBI grading to reflect the influence of portal hypertension. The predictive value of ALBI and PALBI grading has been studied in HCC patients receiving different treatment modality such as c-TACE (8), radiotherapy (9), or hepatectomy (10). However, data on their application are currently insufficient for stage C HCC patients with liver cirrhosis receiving c-TACE. The purpose of this study was to evaluate the prognostic value of PALBI, ALBI, and CP score in stage C HCC patients with liver cirrhosis treated with c-TACE, and to identify the best predictors.

## 2 Materials and methods

**Figure 1:**
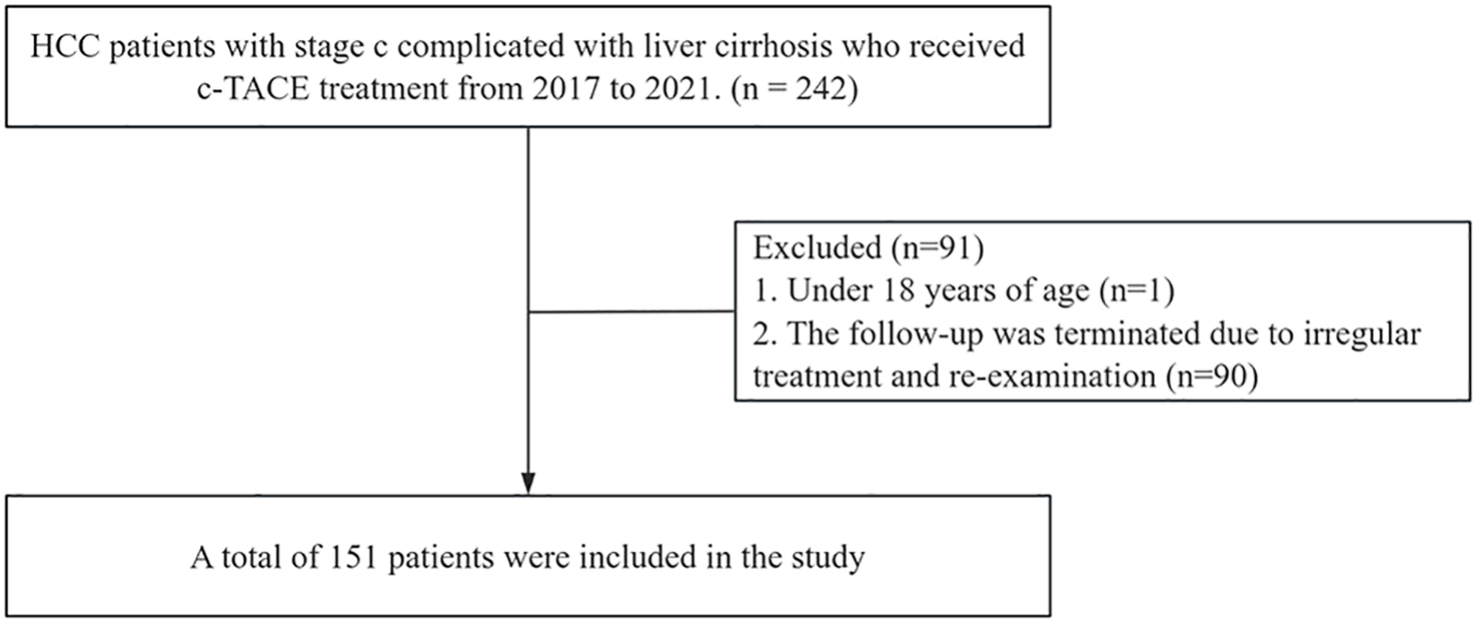
Flow chart of this study.

This study retrospectively included 151 patients with stage C hepatocellular carcinoma with liver cirrhosis who received c-TACE treatment at the Affiliated Hospital of Southwest Medical University between 2017 and 2021 (**Figure 1**).

The inclusion criteria were as follows: histologically or cytologically confirmed HCC; stage C based on the Barcelona Clinic Liver Cancer (BCLC) staging system; liver cirrhosis; Child-Pugh class A or B; Eastern Cooperative Oncology Group performance status (ECOG PS) of 0 to 2; and patients treated with c-TACE.

The exclusion criteria were as follows: the presence of malignancies other than HCC; a history of liver transplantation; patients under 18 years of age; patients with severe complications or comorbidities in other organs, such as (but not limited to) heart failure, respiratory failure, hepatic encephalopathy, abdominal infections, severe hepatic or renal insufficiency; and patients unable to tolerate treatment due to severe adverse reactions. This study was carried out in compliance with the Declaration of Helsinki of the World Medical Association and was approved by the Clinical Trial Ethics Committee of the Affiliated Hospital of Southwest Medical University.

## 3 Therapeutic methods

### 3.1 c-TACE method

The c-TACE procedure is as follows: First, the Seldinger technique is employed to puncture the femoral artery. Subsequently, a catheter is inserted into the hepatic artery for selective angiography, which serves to identify the tumor-feeding artery. A mixture composed of carboplatin, 5-fluorouracil, and papaveretin ethyl iodide is then injected through the catheter into the identified tumor-feeding artery. Additionally, polyvinyl alcohol microballoons are utilized to embolize this artery. Post- embolization, angiography is carried out again. If the angiogram reveals a decrease in the tumor - feeding artery, the c-TACE treatment is considered complete.

### 3.2 Observation method

Follow-up began on the first day after c-TACE treatment. Overall survival (OS) was calculated as the time from the day after c-TACE treatment until the occurrence of death or the time of the last follow-up. The ALBI and PALBI calculation formulas and grading (11) are presented in **Table 1**.

**Table 1:** Formulas and grading of the ALBI and PALBI.

ALBI / PALBI grade	Formula	Grading		
ALBI grade	(log_10_ (Bilirubin (µmol/L)) × 0.66) + (Albumin (g/L) × (−0.085))	ALBI grade 1	ALBI grade 2	ALBI grade 3
		≤−2.6	>−2.6 and ≤−1.39	>−1.39
PALBI grade	2.02 × log_10_ Bilirubin (µmol/L) level − 0.37 × (log_10_ Bilirubin level)^2^ − 0.04 × Albumin level − 3.48 × log_10_ Platelet count (1000/µL) + 1.01 × (log_10_ Platelet count)^2^	PALBI grade 1	PALBI grade 2	PALBI grade 3
		≤−2.53	> −2.53 and ≤−2.09	> −2.09

## 4 Statistics analysis

In this study, case numbers were used to denote the count data, and the chi - square test was applied for its analysis. Measurement data were represented as mean ± standard deviation and analyzed using the T - test. The Cox proportional hazards regression model is used to analyze the risk factors affecting survival rate. The area under the receiver operating characteristic curve (AUROC) is used to compare the predictive value of each indicator to the survival. The Kaplan-Meier method is used to analyze the overall survival (OS), and the log-rank test is used to analyze the significance, with P<0.05 indicating a statistically significant difference. The statistical analysis was performed using SPSS 27.0 software.

## 5 Results

### 5.1 Patient characteristics

In this study, the ages of the patients ranged from 28 to 78 years, with an average age of 54.03 years. There were 133 male patients (88.1%) and 18 female patients (11.9%). Among them, 103 patients (68.2%) were infected with hepatitis B. There were 87 patients (57.6%) with Child-Pugh (CP) class A and 64 patients (42.4%) with class B. The numbers of patients with ALBI grades 1, 2, and 3 were 25 (16.6%), 114 (75.5%), and 12 (7.9%), respectively. The numbers of patients with PALBI grades 1, 2, and 3 were 68 (45.03%), 60 (39.74%), and 23 (15.23%), respectively. A total of 73 patients (48.3%) had portal hypertension, and 65 patients (43%) had ascites. The baseline characteristics of the patients are shown in **Table 2**.

**Table 2:** The baseline characteristics of the patients.

Variables	Patients (N=151)
Age (years), mean ± SD [range]	54.03 ± 10.84 [28–78]
Gender, n (%)	
Male	133 (88.1)
Female	18 (11.9)
HBV infection, n (%)	
Yes	103 (68.2)
No	48 (31.8)
HCV infection, n (%)	
Yes	3 (2)
No	148 (98)
Smoking, n (%)	
Yes	96 (63.6)
No	55 (36.4)
Drinking, n (%)	
Yes	69 (45.7)
No	82 (54.3)
Child-Pugh (CP) grade, n (%)	
A	87 (57.6)
B	64 (42.4)
ALBI grade, n (%)	
grade 1 (≤−2.60)	25 (16.6)
grade 2 (>−2.60 and ≤−1.39)	114 (75.5)
grade 3 (>−1.39)	12 (7.9)
PALBI grade, n (%)	
grade 1 (≤−2.53)	68 (45.03)
grade 2 (> −2.53 and ≤−2.09)	60 (39.74)
grade 3 (> −2.09)	23 (15.23)
Serum AFP level (ng/mL), n (%)	
>400	82 (54.3)
≤400	69 (45.7)
Liver cirrhosis, n (%)	
Yes	151 (100)
Portal hypertension, n (%)	
Yes	73 (48.3)
No	78 (51.7)
Ascites, n (%)	
Yes	65 (43)
No	86 (57)

### 5.2 Survival analysis

**Figure 2:**
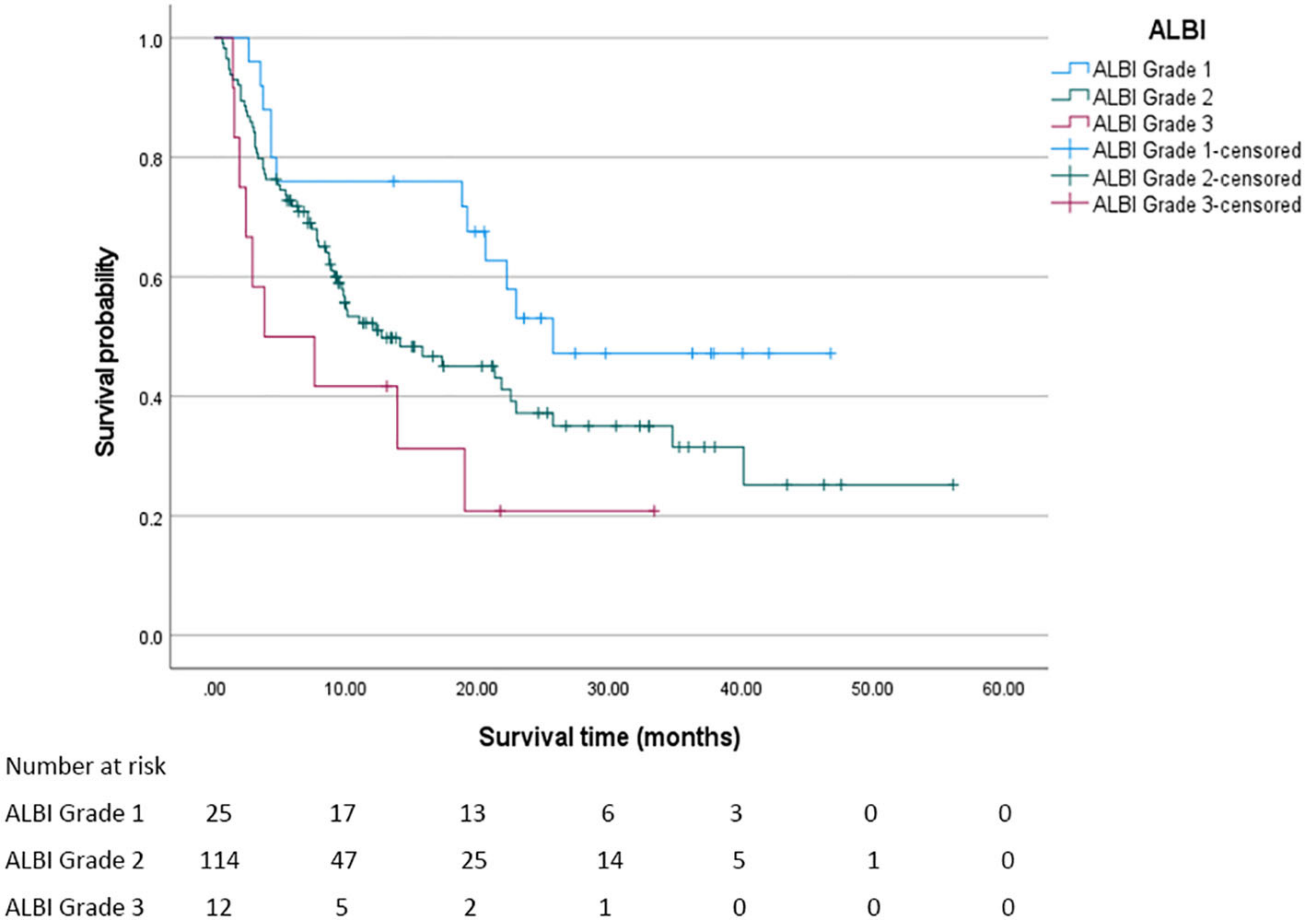
The survival curve of ALBI grading. The median overall survival of patients in each ALBI grade was statistically significant (P < 0.05).

**Figure 3:**
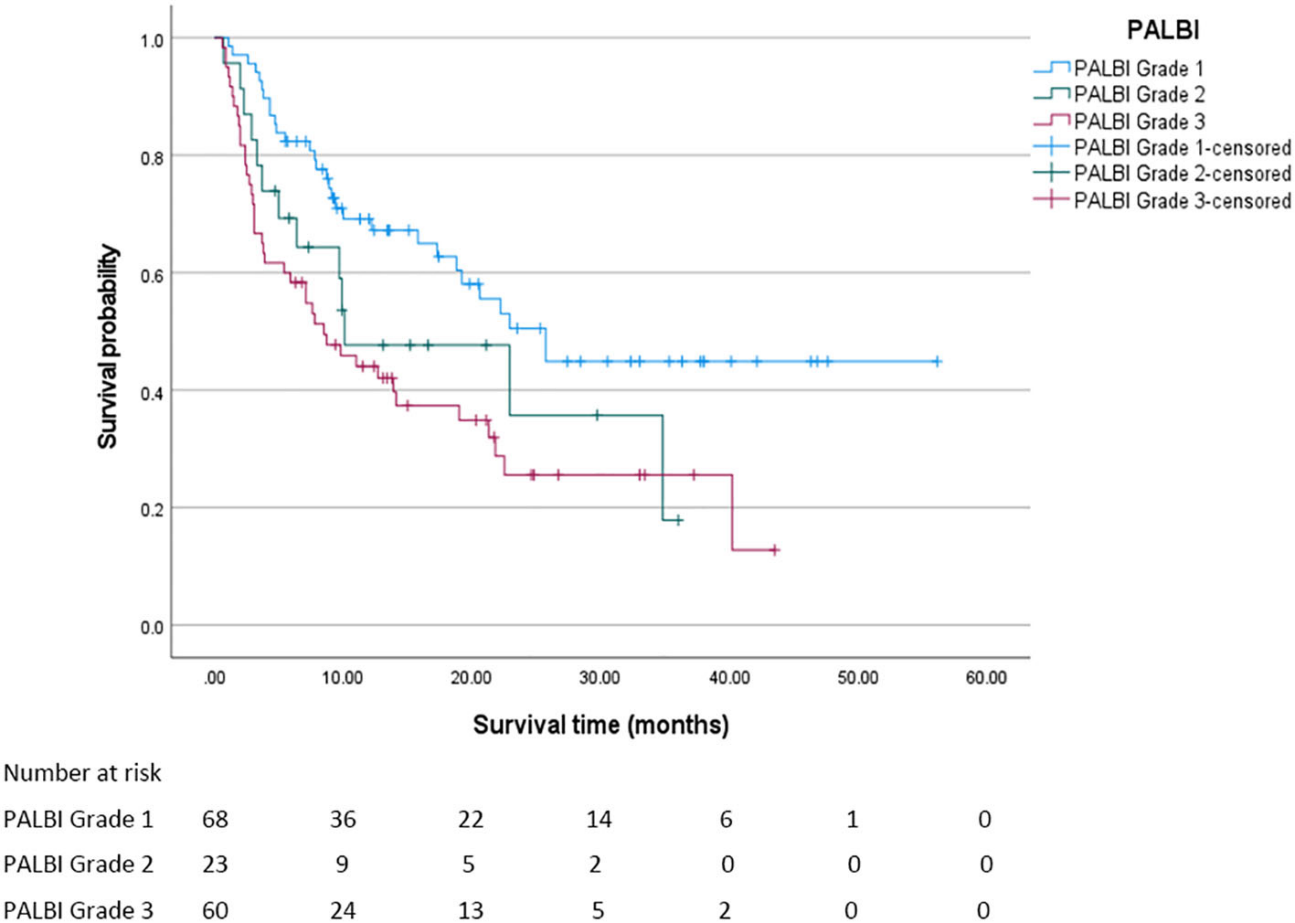
The survival curve of PALBI grading. The median overall survival of patients in each PALBI grade was statistically significant (P < 0.05).

**Figure 4:**
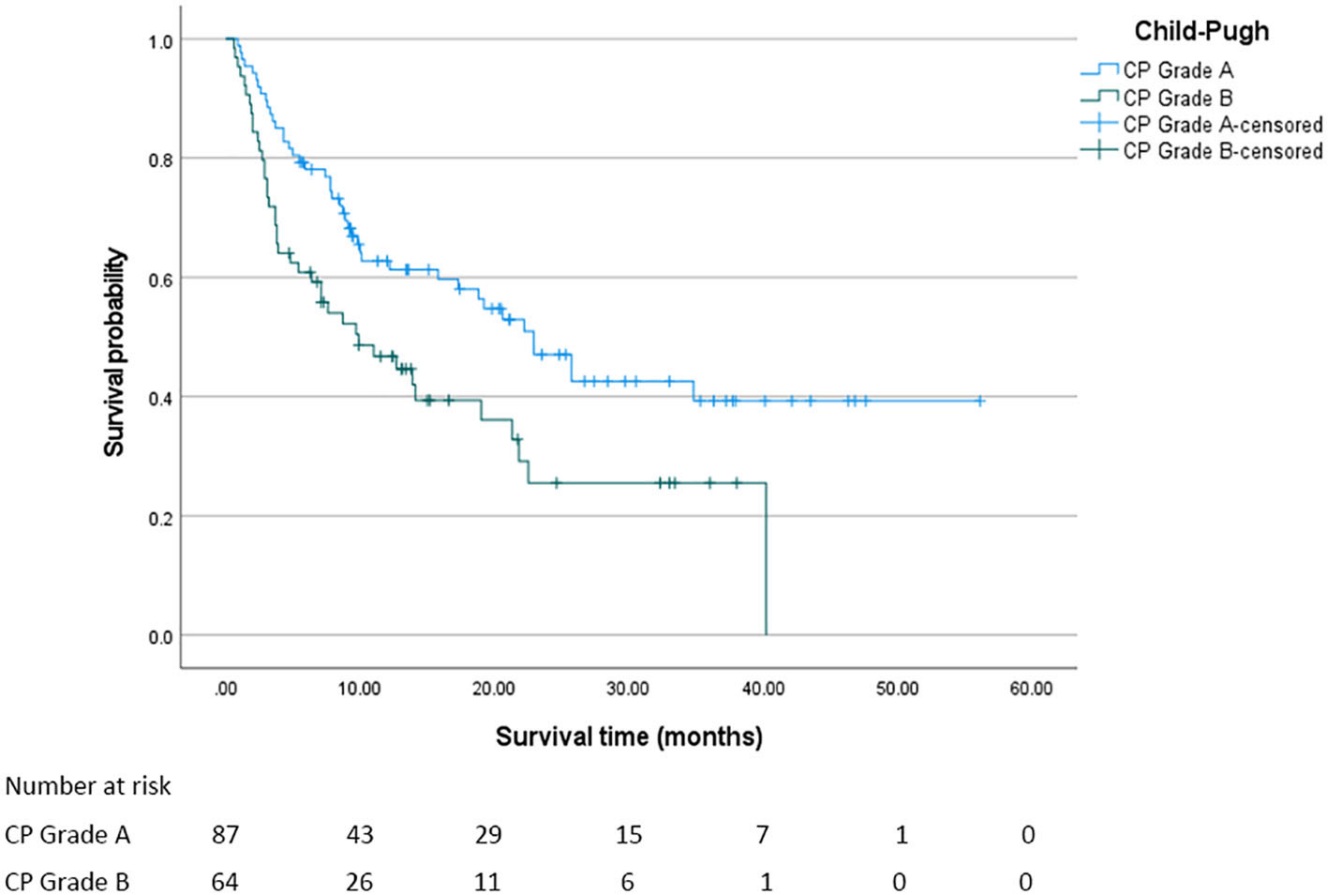
The survival curve of CP grading. The median overall survival of patients in each CP grade was statistically significant (P < 0.05).

In this study, the median overall survival (OS) for the entire cohort was 18.8 months (95% confidence interval [CI]: 11.294 - 26.306 months). Significantly, Child - Pugh (CP) grading, Albumin - Bilirubin (ALBI) grading, and Prognostic Albumin - Bilirubin (PALBI) grading were all associated with OS (all P < 0.05). Regarding ALBI grading, the median OS was 25.7 months for patients in grade 1, 12.7 months for those in grade 2, and 3.8 months for those in grade 3, with a statistically significant difference (P < 0.05). The survival curve based on ALBI grading is depicted in **Figure 2**. For PALBI grading, the median OS was 25.7 months for patients in grade 1, 10.1 months for those in grade 2, and 8.5 months for those in grade 3, showing a statistically significant difference (P < 0.05). The survival curve according to PALBI grading is presented in **Figure 3**. In terms of CP grading, the median OS was 22.9 months for patients in grade A and 9.9 months for those in grade B, with a statistically significant difference (P < 0.05). The survival curve by CP grading is illustrated in **Figure 4**.

### 5.3 Prognostic value of ALBI, PALBI, and CP on survival

**Figure 5:**
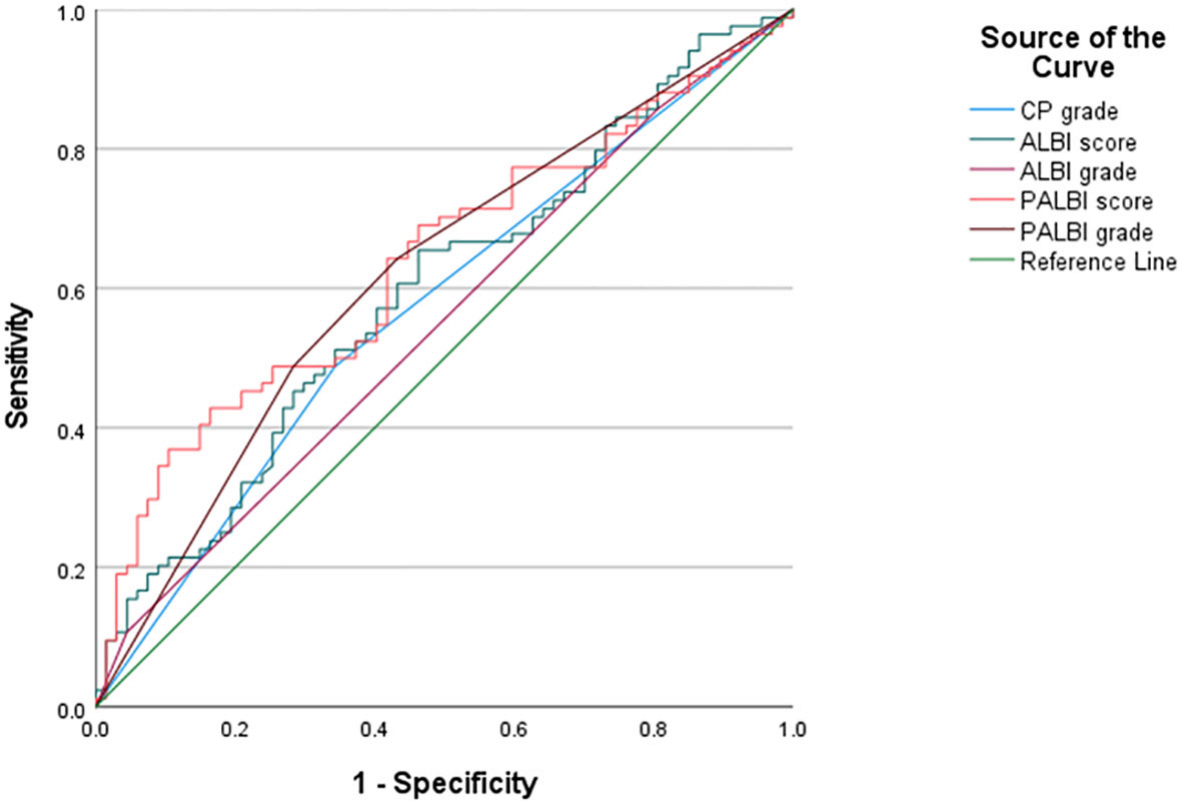
The ROC of CP grade, ALBI grade, ALBI score, PALBI grade and PALBI score. PALBI had a higher the area under the receiver operating characteristic curve than CP and ALBI.

The receiver operating characteristic curve (ROC) analysis was used to evaluate the predictive efficacy of ALBI, PALBI, and CP (**Figure 5**, **Table 3**). The AUROC values of CP grade, ALBI grade, ALBI score, PALBI grade, and PALBI score were 0.572 (95% CI: 0.481-0.664), 0.550 (95% CI: 0.457-0.642), 0.595 (95% CI: 0.505-0.686), 0.619 (95% CI: 0.529-0.709), and 0.637 (95% CI: 0.549-0.725), respectively. In comparison to CP and ALBI, PALBI exhibited superior predictive performance for stage C HCC carcinoma complicated by liver cirrhosis patients who underwent c-TACE treatment.

**Table 3:** AUC value and Harrell’s C-statics for comparing each grades.

Grade / score	Harrell’s C-statistic (95%CI)	P value
CP grade	0.572 (0.481-0.664)	0.122
ALBI grade	0.550 (0.457-0.642)	0.292
ALBI score	0.595 (0.505-0.686)	0.039
PALBI grade	0.619 (0.529-0.709)	0.009
PALBI score	0.637 (0.549-0.725)	0.002

### 5.4 Multivariate Cox analysis

In univariate analysis, CP grade (HR: 1.817, p = 0.007) and portal vein invasion (HR: 1.680, p = 0.028) were associated with decreased survival rate, while the number of c-TACE (HR: 0.685, p = 0.007) was associated with improved survival rate. In multivariate analysis including CP grade, CP grade (HR: 1.704, p = 0.017) and portal vein invasion (HR: 1.916, p = 0.007) were associated with poor prognosis, while the number of c-TACE (HR: 0.668, p = 0.006) was associated with favorable prognosis (**Table 4**).

**Table 4:** Univariate analysis and multivariate analysis of overall survival in patients undergoing transarterial chemoembolization including CP grade.

Prognostic factors	Univariate analysis			Multivariate analysis		
	HR	95% CI	P-value	HR	95% CI	P-value
CP grade (Class A vs. Class B)	1.817	1.179-2.801	0.007	1.704	1.101-2.638	0.017
Portal vein invasion (yes vs. no)	1.680	1.059-2.667	0.028	1.916	1.198-3.063	0.007
Portal hypertension (yes vs. no)	0.687	0.444-1.062	0.091			
Intrahepatic metastasis (yes vs. no)	1.068	0.640-1.783	0.800			
Ascites (yes vs. no)	1.387	0.901-2.134	0.137			
Hepatitis B (yes vs. no)	1.226	0.763-1.969	0.400			
Hepatitis C (yes vs. no)	1.350	0.331-5.512	0.676			
Smoking history (yes vs. no)	1.556	0.979-2.473	0.062			
Drinking history (yes vs. no)	0.917	0.595-1.412	0.693			
The number of c-TACE	0.685	0.521-0.900	0.007	0.668	0.499-0.893	0.006
The level of AFP (> 400 vs. ≦ 400)	1.123	0.731-1.725	0.598			
ECOG PS	0.925	0.675-1.267	0.628			
Number of tumors (> 3 vs. ≦ 3)	1.298	0.777-2.166	0.319			
Tumor diameter (> 5 vs. ≦ 5cm)	1.437	0.887-2.328	0.141			

In univariate analysis, ALBI grade (HR: 1.731, p = 0.013) and portal vein invasion (HR: 1.680, p = 0.028) were associated with decreased survival rate, while the number of c-TACE (HR: 0.685, p = 0.007) was associated with improved survival rate. In multivariate analysis including ALBI grade, ALBI grade (HR: 1.573, p = 0.045) and portal vein invasion (HR: 1.866, p = 0.009) were associated with poor prognosis, while the number of c-TACE (HR: 0.685, p = 0.007) was associated with good prognosis (**Table 5**).

**Table 5:** Univariate analysis and multivariate analysis of overall survival in patients undergoing transarterial chemoembolization including ALBI grade.

Prognostic factors	Univariate analysis			Multivariate analysis		
	HR	95% CI	P-value	HR	95% CI	P-value
ALBI grade (grade 1 vs. grade 2 vs. grade 3)	1.731	1.122-2.669	0.013	1.573	1.011-2.446	0.045
Portal vein invasion (yes vs. no)	1.680	1.059-2.667	0.028	1.866	1.169-2.978	0.009
Portal hypertension (yes vs. no)	0.687	0.444-1.062	0.091			
Intrahepatic metastasis (yes vs. no)	1.068	0.640-1.783	0.800			
Ascites (yes vs. no)	1.387	0.901-2.134	0.137			
Hepatitis B (yes vs. no)	1.226	0.763-1.969	0.400			
Hepatitis C (yes vs. no)	1.350	0.331-5.512	0.676			
Smoking history (yes vs. no)	1.556	0.979-2.473	0.062			
Drinking history (yes vs. no)	0.917	0.595-1.412	0.693			
The number of c-TACE	0.685	0.521-0.900	0.007	0.685	0.519-0.904	0.007
The level of AFP (> 400 vs. ≦ 400)	1.123	0.731-1.725	0.598			
ECOG PS	0.925	0.675-1.267	0.628			
Number of tumors (> 3 vs. ≦ 3)	1.298	0.777-2.166	0.319			
Tumor diameter (> 5 vs. ≦ 5cm)	1.437	0.887-2.328	0.141			

In univariate analysis, PALBI grade (HR: 1.482, p = 0.001) and portal vein invasion (HR: 1.680, p = 0.028) were associated with decreased survival rate, while the number of c-TACE (HR: 0.685, p = 0.007) was associated with increased survival rate. In multivariate analysis including PALBI grade, PALBI grade (HR: 1.366, p = 0.012) and portal vein invasion (HR: 1.825, p = 0.011) were associated with poor prognosis, while the number of c-TACE (HR: 0.704, p = 0.016) was associated with good prognosis (**Table 6**).

**Table 6:** Univariate analysis and multivariate analysis of overall survival in patients undergoing transarterial chemoembolization including PALBI grade.

Prognostic factors	Univariate analysis			Multivariate analysis		
	HR	95% CI	P-value	HR	95% CI	P-value
PALBI grade (grade 1 vs. grade 2 vs. grade 3)	1.482	1.173-1.873	0.001	1.366	1.071-1.742	0.012
Portal vein invasion (yes vs. no)	1.680	1.059-2.667	0.028	1.825	1.147-2.904	0.011
Portal hypertension (yes vs. no)	0.687	0.444-1.062	0.091			
Intrahepatic metastasis (yes vs. no)	1.068	0.640-1.783	0.800			
Ascites (yes vs. no)	1.387	0.901-2.134	0.137			
Hepatitis B (yes vs. no)	1.226	0.763-1.969	0.400			
Hepatitis C (yes vs. no)	1.350	0.331-5.512	0.676			
Smoking history (yes vs. no)	1.556	0.979-2.473	0.062			
Drinking history (yes vs. no)	0.917	0.595-1.412	0.693			
The number of c-TACE	0.685	0.521-0.900	0.007	0.704	0.529-0.937	0.016
The level of AFP (> 400 vs. ≦ 400)	1.123	0.731-1.725	0.598			
ECOG PS	0.925	0.675-1.267	0.628			
Number of tumors (> 3 vs. ≦ 3)	1.298	0.777-2.166	0.319			
Tumor diameter (> 5 vs. ≦ 5cm)	1.437	0.887-2.328	0.141			

## 6 Discussion

In this study, we performed c-TACE therapy on patients with stage C hepatocellular carcinoma complicated with cirrhosis and obtained a series of valuable results. The median overall survival (OS) of the entire study cohort was 18.8 months, which provides an important reference for evaluating the overall survival of this patient population. Through further analysis, we found that CP score, ALBI grade and PALBI grade were all significantly correlated with patients’ OS, which clarified the importance of these three scoring systems in predicting patients’ prognosis.

The research findings of Soon Kyu Lee et al. indicated that the PALBI and ALBI grades could serve as predictors of the OS in patients with HCC (12). Similarly, Jan Hansmann et al. conducted research and discovered that the PALBI and ALBI grades were capable of predicting the overall survival of high - risk patients with HCC who underwent c-TACE treatment (8). This is in line with the results of our current study.

Although the subjects in this study were liver cancer patients with cirrhosis, the tumor prognosis of advanced liver cancer is mainly closely related to liver reserve, and liver function models, such as ALBI and CP scores, have clinically relevant prognostic effects in systemic treatment of advanced HCC (13). Even with cirrhosis, as long as the liver function reserve is good, you can live with a better prognosis after treatment.

In this study, the grading of these three scoring systems can effectively distinguish the prognosis of patients, and different grades of each scoring system have significant differences in the impact of patients’ median OS. In the ALBI grading system, as the grade progresses from 1 to 3, the median OS of patients plummets drastically from 25.7 months to 3.8 months. This vividly demonstrates that as the liver function deteriorates from a relatively sound state to a severely impaired one, the survival time of patients is significantly curtailed. Likewise, in the PALBI grading, the median OS of patients at different levels also exhibits a clear - cut gradient change. Patients at grade 1 enjoy a distinct survival advantage, while as the grade goes up, the survival time gradually diminishes. In CP grading, the median OS of grade A patients was also significantly longer than grade B patients. The OS of PALBI grade 3 patients (8.5 months) was higher than that of ALBI grade 3 patients (3.8 months). This phenomenon may be closely related to the design logic of the two scoring systems, the differences in patients’ tolerance and response to c-TACE treatment, and the impact of complications. The ALBI score is based only on albumin and bilirubin, focusing on liver synthetic function and cholestasis. Grade 3 usually corresponds to severe hypoproteinemia and hyperbilirubinemia, directly reflecting severe impairment of liver metabolism and synthetic function. In contrast, the PALBI score incorporates platelet count in addition to albumin and bilirubin, providing a more comprehensive assessment of the dual damage of “liver function + portal hypertension” caused by liver cirrhosis. The efficacy of c-TACE depends on liver reserve function to tolerate ischemic injury and recover. c-TACE treatment works by embolizing the tumor-supplying arteries. During the process, it may cause ischemic injury to hepatocytes, and the liver’s regenerative ability is closely related to prognosis. Platelets are not only key components of coagulation function, but also participate in the repair of hepatic sinusoidal endothelial cells and hepatocyte regeneration by releasing cytokines such as platelet-derived growth factor (PDGF) and transforming growth factor-β (TGF-β) (14). After liver injury, platelets are recruited to the liver, which may be related to Kupffer cells. Growth factors in platelets, such as IGF-1 and HGF, activate the Akt and ERK1/2 pathways and have a proliferative effect on liver cells. Platelets activate liver cell proliferation through the activation of liver sinusoidal endothelial cells (LSEC). The direct contact between platelets and LSEC triggers the secretion of sphingosine 1-phosphate (S1P) by platelets, thereby inducing LSEC to secrete IL-6; subsequently, the increase in IL-6 leads to the activation of the STAT3 pathway, the activation of Akt and ERK1/2, and the promotion of liver cell proliferation (15–18). For patients with relatively good platelet indicators in the PALBI score, platelets after c-TACE may promote liver tissue repair and alleviate ischemic liver injury through the above mechanism. Patients with ALBI grade 3 May suffer from insufficient platelet function or quantity, lacking adequate regenerative signal support, leading to continuous aggravation of liver damage and a shortened survival period. Future studies can further monitor the dynamic changes of platelet count after c-TACE (such as the levels at 1 week, 1 month, and 3 months), analyze its correlation with the recovery trend of liver function indicators (such as Alaninetransaminase, Aspartate aminotransferase, Albumin), and clarify the specific role of platelets in liver injury repair. ALBI grade 3 patients may have more severe synthetic function failure, resulting in a worse baseline condition. PALBI grade 3 patients may have poor liver reserve function, but not to the extreme degree of ALBI grade 3. After embolization, tumor necrosis occurs, but the remaining liver tissue can still maintain basic function. In contrast, ALBI grade 3 patients have severely decompensated livers, and c-TACE may induce irreversible liver failure, accelerating disease progression. The comprehensive assessment of “liver function + portal hypertension” by the PALBI score may more accurately screen out patients suitable for c-TACE treatment - even grade 3 patients have a better baseline condition and treatment tolerance than ALBI grade 3 patients, and thus achieve a longer survival period after c-TACE. This suggests that in liver cancer patients with liver cirrhosis, the PALBI score may be more suitable than the ALBI score for guiding c-TACE treatment decisions and prognosis assessment. In our study, there were only 12 patients with ALBI grade 3, accounting for 7.9% of the total sample size. The small sample size of this subgroup may have a certain impact on the stability and interpretability of the results. From a statistical perspective, the survival analysis results of small sample subgroups are susceptible to interference from extreme values or random errors, which may lead to an overestimation of survival differences. For instance, the 3.8-month survival period of ALBI grade 3 patients observed in this study may have a relatively wide 95% confidence interval, and its actual clinical significance needs to be interpreted with caution. This phenomenon is not uncommon in clinical research, especially for subgroups with more severe conditions and lower incidence rates. For instance, ALBI grade 3 indicates severe liver function impairment, and the difficulty in expanding the sample size is often an objective limitation. However, it should be made clear that such results are more of exploratory significance rather than definite conclusions. To verify its reliability, it is recommended that further verification be carried out through sensitivity analysis in the future. For instance, different statistical models can be adopted, such as the correction method for small samples in the Cox proportional hazards model, or re-analysis can be conducted after excluding extreme values to evaluate the stability of the results. At the same time, when interpreting the survival data of this subgroup, its limitations should be clearly marked to avoid excessive inference. Future research can increase the number of ALBI grade 3 patients included through multi-center collaboration, expanding the sample size and other methods in a targeted manner to obtain more representative results, thereby more accurately revealing the survival patterns and clinical characteristics of this group.

The multivariate analysis results of this study indicated that CP grade, ALBI grade, PALBI grade, portal vein invasion, and the number of c-TACE treatments were all independent predictors of the prognosis for HCC patients with liver cirrhosis who received c-TACE treatment. This finding reveals that on the basis of liver cirrhosis, the biological characteristics of the disease itself, the state of liver function reserve, and the intensity of treatment intervention jointly constitute the key dimensions that affect the prognosis for HCC patients receiving local treatment. The clinical significance of this finding is worthy of in-depth analysis. Portal vein invasion, as an independent predictive factor, once again confirms the core role of tumor invasiveness in the progression of liver cancer in liver cirrhosis. In the context of liver cirrhosis, the vascular structure within the liver is distorted due to fibrosis, making the portal venous system more likely to serve as a channel for tumor cell infiltration and metastasis. Once invasion occurs, it not only indicates that the biological behavior of the tumor has a more malignant potential, but also may exacerbate liver function damage by blocking blood flow, increase the risk of intrahepatic recurrence, and thereby weaken the local control effect of c-TACE treatment. In a study, it was also suggested that portal vein invasion might be the most important risk factor for immediate recurrence after HCC surgery (19). The independent prognostic value of the number of c-TACE treatments is of particular significance in liver cancer patients with liver cirrhosis. For liver cancer patients with liver cirrhosis, the number of c-TACE treatments not only reflects the tumor’s response to treatment (such as the need for multiple treatments to control progressive lesions), but is also closely related to liver function tolerance. CP grade, ALBI grade, PALBI grade all demonstrated independent prognostic value in the multivariate analysis, highlighting the decisive influence of liver function reserve in the context of liver cirrhosis on the treatment outcomes of c-TACE patients. The five independent predictive factors identified in this study have constructed a prognostic assessment system for liver cancer patients with liver cirrhosis who receive c-TACE treatment from three aspects: portal vein invasion reflects the invasive potential of the tumor in the context of liver cirrhosis, the number of c-TACE treatments represents the “dosage-effect balance” of the therapeutic intervention, while the Child-Pugh, ALBI, and PALBI scores reveal the fundamental impact of liver function reserve in cirrhotic patients on treatment tolerance and survival. In clinical practice, these factors need to be integrated into treatment decisions. Future research can further explore the interaction of these factors (such as the determination of the “optimal treatment frequency” based on liver function indicators), to provide more precise guidance for individualized c-TACE treatment for liver cancer patients with liver cirrhosis. Multivariate analysis showed that the tumor marker AFP was not an independent risk factor. Liver cirrhosis itself could lead to liver cell regeneration and inflammatory activity, thereby causing a mild increase in AFP. This non-tumor increase might dilute the association between AFP and the malignancy of the tumor. The efficacy of c-TACE mainly depends on the local characteristics of the tumor (size, number, blood supply), the tolerance of liver function in the context of liver cirrhosis (whether multiple treatments can be tolerated), and the degree of tumor necrosis after treatment. Therefore, compared with AFP, liver function status, portal vein invasion and the number of c-TACE treatments may have a more direct impact on the outcome and become more critical independent risk factors.

Theodora Oikonomou et al. found that ALBI and PALBI grades can be used to predict the prognosis of patients with stable decompensated cirrhosis. Albi and Palbi performed better than CP scores in the study (20). However, in a study of cirrhosis, ALBI had the best sensitivity and specificity balance (AUC 0.704, 95%CI 0.630 ~ 0.778) compared with Child-Pugh and PALBI scores, and ALBI score may be a better prognostic indicator of cirrhosis mortality (21). This is inconsistent with our research results. This difference may reflect that the predictive efficacy of the liver function scoring tool is influenced by multiple factors such as the characteristics of the study population and the mode of treatment intervention. Our study population consisted of patients with HCC and liver cirrhosis. HCC is often accompanied by more complex pathological and physiological changes: tumor occupation leads to intrahepatic blood flow disorder, portal vein tumor thrombus formation aggravates portal hypertension, and tumor metabolites affect liver synthetic function, etc. At the same time, c-TACE treatment will further exacerbate liver microcirculation disorders and portal hypertension through mechanisms such as “blocking tumor blood supply arteries”, “chemotherapy drug toxicity”, and “ischemia-reperfusion injury”. The PALBI score incorporates platelet count on the basis of ALBI, and platelets reflect the degree of portal hypertension and the state of splenomegaly. This precisely captures the key prognostic information under the interaction of “tumor - liver cirrhosis - treatment damage” in these patients, such as the risk of portal hypertension-related bleeding and the risk of acute deterioration of liver reserve function. Therefore, the predictive efficacy is better. In the study by Fragaki (21) et al. on the liver cirrhosis population, liver function impairment was mainly driven by the progression of liver fibrosis. The ALBI score only relied on albumin and bilirubin, directly reflecting the reserve capacity of liver parenchymal cells, while the core driving factor of liver cirrhosis mortality is the failure of liver parenchymal function. Therefore, ALBI is more capable of accurately anchoring the risk of death in this population. The conflict between the results of the two studies is not an absolute judgment of “score superiority or inferiority”, but rather suggests that the selection of liver function scoring should be closely combined with the patient’s disease characteristics and treatment methods.

In this study, when comparing the predictive capabilities of each scoring system, by means of the crucial indicator - AUROC, we unearthed more significant insights. The AUROC values of the CP score, ALBI grade, ALBI score, PALBI grade, and PALBI score vary. Among them, the AUROC values associated with PALBI are relatively high. The ROC analysis showed that only the two indicators related to PALBI had statistically significant differences, indicating that PALBI is more capable of reflecting the prognosis of HCC with liver cirrhosis treated by c-TACE. This datum reveals that in contrast to the CP score and ALBI, PALBI exhibits superior efficacy in predicting the prognosis of patients with stage C HCC complicated by liver cirrhosis who have received c-TACE treatment. Similarly, for HCC patients who received radiotherapy, the AUC value of the PALBI grade is higher than that of the ALBI grade and the CP score. Both the PALBI and ALBI grades can estimate the survival rate more precisely than the CP score (9). This indicates that the PALBI grade indeed has an edge in predicting the prognosis of HCC patients with cirrhosis. In this study, the AUROC values of ALBI grade and PALBI grade were lower than those of ALBI score and PALBI score. Then, can classification scores completely replace continuous scores? The grading of ALBI and PALBI can to some extent replace their continuous scores for evaluating liver function and related prognosis, but not in all cases can they completely replace. Continuous ALBI and PALBI scores provide a more nuanced representation of liver function. They can detect subtle changes in liver function that may be missed by the grading systems. For instance, small fluctuations in albumin, bilirubin, and pre - albumin levels can be accurately reflected in the continuous scores, while these changes may not lead to a change in the grade. The grading systems of ALBI and PALBI, while being convenient for quick clinical assessment, have limitations. They group patients into a few categories, which may overlook the individual differences in liver function. As a result, the AUC values of the grading systems are lower compared to the continuous scores. In clinical practice, the choice between continuous scores and grading systems should be based on the specific situation. For patients with stable conditions, the grading systems can be used for initial assessment. In contrast, for patients with rapidly changing conditions, continuous scores are more appropriate for closely monitoring liver function changes.

Nevertheless, we must be acutely aware that despite the strong support this study provides for the advantageous position of PALBI in predicting the prognosis of such patients, there are still certain limitations. Firstly, platelet count is influenced by multiple factors, and its change mechanism under diverse disease conditions is intricate, which may exert a certain influence on the stability of the PALBI score. Secondly, the broad applicability of the results of this study still requires further verification through more large - scale, multi - center studies. Therefore, larger scale, multi-center research is very necessary. Finally, this study did not conduct a correlation study on the predictive effect of each scoring system on treatment complications, which may also be needed in subsequent studies. This study found that the AUROC values of ALBI, PALBI and Child scores were all low. This result suggests that the above liver function-related scores have limited discriminatory ability for the clinical outcomes of this population, such as treatment response, postoperative complications or survival prognosis. According to the explanation of AUROC, the values in the 0.5 to 0.7 range indicate that the discriminatory ability is only slightly better than random judgment, and the performance of each score in this study falls within this range. This may be related to the particularity of c-TACE treatment and the complexity of the patient’s condition: c-TACE therapy exerts its effect by embolizing tumor blood vessels. Its efficacy and safety not only depend on the basal liver function reserve, but are also closely related to multiple factors such as tumor burden (such as size, number, vascular invasion), treatment plan (such as embolization degree, drug selection), and the overall condition of the patient. A single liver function score is difficult to comprehensively capture the interaction of these complex variables, and thus its independent predictive value is limited. Although their discriminatory ability is limited when applied alone, these scores may still have certain reference value in clinical practice. However, their application scenarios need to be further optimized in combination with the combined strategy. Tumor markers related to liver cancer (such as PIVKA-II) can reflect the biological behavior of tumors (such as proliferative activity and invasiveness), while scores like PALBI focus on liver function status. The combination of the two can achieve multi-dimensional assessment of “tumor characteristics - liver function reserve”. For example, when predicting tumor progression after c-TACE, patients with good liver function tolerance can be screened through PALBI scores, etc. Combined with the tumor active status indicated by high PIVKA-II levels, populations at high risk of progression can be identified more accurately, providing a basis for subsequent treatment adjustments (such as shortening the re-examination interval or combining targeted drugs). It should be admitted that the current results suggest that relying solely on the above liver function scores is difficult to meet the demand for precise prediction. Just like the GALAD score (22), Future research can further explore the construction of a combined model For example, tumor markers (PIVKA-II), ALBI, PALBI, Child scores, imaging features (such as tumor necrosis rate), and treatment-related indicators (such as embolic agent dosage) were included. The predictive model was optimized through multivariate analysis to enhance the ability to distinguish the treatment outcomes of c-TACE. Meanwhile, the particularity of this population also needs to be considered, and a “liver function - tumor characteristics” joint assessment system that is more suitable for the c-TACE treatment scenario should be developed specifically to better serve clinical decision-making.

In summary, within the current research context, for patients with stage C HCC complicated by liver cirrhosis who have received c-TACE treatment, the PALBI grade has demonstrated conspicuous advantages in predicting the prognosis and is anticipated to become a potent tool for clinicians to assess the prognosis of patients and formulate personalized treatment plans. However, when using the PALBI grade, it is necessary to combine whether there is portal vein invasion and the number of c-TACE treatments for comprehensive evaluation, as well as to further explore and construct a combined model. To improve its application effect in clinical practice, further in-depth research is still needed.

## Data Availability

The original contributions presented in the study are included in the article/supplementary material. Further inquiries can be directed to the corresponding authors.

## References

[1] Bray F Ferlay J Soerjomataram I Siegel RL Torre LA Jemal A. Global cancer statistics 2018: GLOBOCAN estimates of incidence and mortality worldwide for 36 cancers in 185 countries. CA Cancer J Clin. (2018) 68:394–424. doi: 10.3322/caac.21492, PMID: 30207593

[2] European Association for the Study of the Liver. EASL Clinical Practice Guidelines: Management of hepatocellular carcinoma. J Hepatol. (2018) 69:182–236. doi: 10.1016/j.jhep.2018.03.019, PMID: 29628281

[3] Heimbach JK Kulik LM Finn RS Sirlin CB Abecassis MM Roberts LR. AASLD guidelines for the treatment of hepatocellular carcinoma. Hepatology. (2018) 67:358–80. doi: 10.1002/hep.29086, PMID: 28130846

[4] Kaneko S Tsuchiya K Yasui Y Inada K Kirino S Yamashita K. Strategy for advanced hepatocellular carcinoma based on liver function and portal vein tumor thrombosis. Hepatol Res. (2020) 50:1375–85. doi: 10.1111/hepr.13567, PMID: 32924266

[5] Cucarull B Tutusaus A Rider P Hernáez-Alsina T Cuño C García de Frutos P. Hepatocellular carcinoma: molecular pathogenesis and therapeutic advances. Cancers (Basel). (2022) 14:621. doi: 10.3390/cancers14030621, PMID: 35158892 PMC8833604

[6] Peng Y Qi X Guo X. Child-pugh versus MELD score for the assessment of prognosis in liver cirrhosis: A systematic review and meta-analysis of observational studies. Med (Baltimore). (2016) 95:e2877. doi: 10.1097/MD.0000000000002877, PMID: 26937922 PMC4779019

[7] Johnson PJ Berhane S Kagebayashi C Satomura S Teng M Reeves HL. Assessment of liver function in patients with hepatocellular carcinoma: a new evidence-based approach-the ALBI grade. J Clin Oncol. (2015) 33:550–8. doi: 10.1200/JCO.2014.57.9151, PMID: 25512453 PMC4322258

[8] Hansmann J Evers MJ Bui JT Lokken RP Lipnik AJ Gaba RC. Albumin-bilirubin and platelet-albumin-bilirubin grades accurately predict overall survival in high-risk patients undergoing conventional transarterial chemoembolization for hepatocellular carcinoma. J Vasc Interv Radiol. (2017) 28:1224–1231.e2. doi: 10.1016/j.jvir.2017.05.020, PMID: 28688815

[9] Ho CHM Chiang CL Lee FAS Choi HCW Chan JCH Yeung CSY. Comparison of platelet-albumin-bilirubin (PALBI), albumin-bilirubin (ALBI), and child-pugh (CP) score for predicting of survival in advanced hcc patients receiving radiotherapy (RT). Oncotarget. (2018) 9:28818–29. doi: 10.18632/oncotarget.25522, PMID: 29988960 PMC6034750

[10] Luo HM Zhao SZ Li C Chen LP. Preoperative platelet-albumin-bilirubin grades predict the prognosis of patients with hepatitis B virus-related hepatocellular carcinoma after liver resection: A retrospective study. Med (Baltimore). (2018) 97:e0226. doi: 10.1097/MD.0000000000010226, PMID: 29561452 PMC5895341

[11] Ho SY Liu PH Hsu CY Huang YH Liao JI Su CW. Comparison of four albumin-based liver reserve models (ALBI/EZ-ALBI/PALBI/PAL) against MELD for patients with hepatocellular carcinoma undergoing transarterial chemoembolization. Cancers (Basel). (2023) 15:1925. doi: 10.3390/cancers15071925, PMID: 37046586 PMC10093004

[12] Lee SK Song MJ Kim SH Park M. Comparing various scoring system for predicting overall survival according to treatment modalities in hepatocellular carcinoma focused on Platelet-albumin-bilirubin (PALBI) and albumin-bilirubin (ALBI) grade: A nationwide cohort study. PloS One. (2019) 14:e0216173. doi: 10.1371/journal.pone.0216173, PMID: 31048923 PMC6497276

[13] da Fonsecaa LG de Melob MAZ da Silveirac THM Yamamotod VJ Hashizumee PHS Sabbagaf J. Prognostic role of albumin-bilirubin (ALBI) score and Child-Pugh classification in patients with advanced hepatocellular carcinoma under systemic treatment. Ecancermedicalscience. (2024) 18:1748. doi: 10.3332/ecancer.2024.1748, PMID: 39421189 PMC11484683

[14] Nowatari T Fukunaga K Ohkohchi N. Regulation of signal transduction and role of platelets in liver regeneration. Int J Hepatol. (2012) 2012:542479. doi: 10.1155/2012/542479, PMID: 22811921 PMC3395153

[15] Murata S Matsuo R Ikeda O Myronovych A Watanabe M Hisakura K. Platelets promote liver regeneration under conditions of Kupffer cell depletion after hepatectomy in mice. World J Surg. (2008) 32:1088–96. doi: 10.1007/s00268-008-9493-0, PMID: 18311501

[16] Kawasaki T Murata S Takahashi K Nozaki R Ohshiro Y Ikeda N. Activation of human liver sinusoidal endothelial cell by human platelets induces hepatocyte proliferation. J Hepatol. (2010) 53:648–54. doi: 10.1016/j.jhep.2010.04.021, PMID: 20615569

[17] Meyer J Lejmi E Fontana P Morel P Gonelle-Gispert C Bühler L. A focus on the role of platelets in liver regeneration: Do platelet-endothelial cell interactions initiate the regenerative process? J Hepatol. (2015) 63:1263–71. doi: 10.1016/j.jhep.2015.07.002, PMID: 26169159

[18] Takahashi K Murata S Ohkohchi N. Novel therapy for liver regeneration by increasing the number of platelets. Surg Today. (2013) 43:1081–7. doi: 10.1007/s00595-012-0418-z, PMID: 23180116

[19] Choi KK Kim SH Choi SB Lim JH Choi GH Choi JS. Portal venous invasion: the single most independent risk factor for immediate postoperative recurrence of hepatocellular carcinoma. J Gastroenterol Hepatol. (2011) 26:1646–51. doi: 10.1111/j.1440-1746.2011.06780.x, PMID: 21592228

[20] Oikonomou T Goulis L Doumtsis P Tzoumari T Akriviadis E Cholongitas E. ALBI and PALBI grades are associated with the outcome of patients with stable decompensated cirrhosis. Ann Hepatol. (2019) 18:126–36. doi: 10.5604/01.3001.0012.7904, PMID: 31113581

[21] Fragaki M Sifaki-Pistolla D Orfanoudaki E Kouroumalis E. Comparative evaluation of ALBI, MELD, and Child-Pugh scores in prognosis of cirrhosis: is ALBI the new alternative? Ann Gastroenterol. (2019) 32:626–32. doi: 10.20524/aog.2019.0417, PMID: 31700241 PMC6826070

[22] Shahini E Pasculli G Solimando AG Tiribelli C Cozzolongo R Giannelli G. Updating the clinical application of blood biomarkers and their algorithms in the diagnosis and surveillance of hepatocellular carcinoma: A critical review. Int J Mol Sci. (2023) 24:4286. doi: 10.3390/ijms24054286, PMID: 36901717 PMC10001986

